# Correlation between Preoperative Platelet Count/(Lymphocyte Count × Prealbumin Count) Ratio and the Prognosis of Patients with Gastric Cancer Undergoing Radical Operation

**DOI:** 10.1155/2023/8401579

**Published:** 2023-07-28

**Authors:** Yi Liu, Yanguang Yang, Guomei Tai, Feng Ni, Cenming Yu, Wenjing Zhao, Ding Wang

**Affiliations:** ^1^Department of Radiotherapy, Affiliated Tumor Hospital of Nantong University, Nantong Tumor Hospital, Nantong, Jiangsu, China; ^2^Cancer Research Center Nantong, Affiliated Tumor Hospital of Nantong University, Nantong Tumor Hospital, Nantong, Jiangsu, China; ^3^Department of Gastrointestinal Surgery, Affiliated Tumor Hospital of Nantong University, Nantong Tumor Hospital, Nantong, Jiangsu, China

## Abstract

**Objective:**

To clarify the relationship between preoperative platelet count/(lymphocyte count × prealbumin count) ratio (PLPR) and the prognosis of patients with gastric cancer undergoing a radical operation, combined with Tumor Node Metastasis (TNM) staging, a scoring system was established to guide clinical application.

**Methods:**

The clinical data of 238 patients receiving radical operations for gastric cancer were retrospectively analyzed. According to the area under the Receiver operating characteristic curve, the predictive value of the preoperative PLPR for the 5-year overall survival (OS) of gastric cancer was determined, and the best cut-off value of the ratio was corresponding to the maximum value of Yoden index. Chi-squared test was applied to analyze the correlation between the ratio and clinicopathological features. Kaplan–Meier curve was applied to analyze the influence of this ratio on 5-year OS. The Cox regression model was applied to analyze the hazards affecting the long-term survival of patients. The nomogram model was used to predict the long-term survival rate.

**Results:**

The optimal cut-off point of preoperative PLPR ratio was 7.46, and the patients were segmented into two sets: one set of ratio <7.46 and another set of ratio ≥7.46. The ratio was correlated with the size of the tumor, T stage, N stage, total stage, vascular cancer thrombus, and nerve invasion. In stage I–III patients, the prognosis was better in the low-ratio set than in the high-ratio set (*P* < 0.001), subgroup analysis indicated the prognosis was obviously better in the low-ratio set than in the high-ratio set in stage II and III patients (*P* < 0.05 and *P* < 0.001), but there was no difference in stage I patients (*P* > 0.05). Age, T stage, N stage, total TNM stage, tumor size, vascular tumor thrombus, nerve invasion, preoperative neutrophil count/lymphocyte count (NLR; reference value 3.68), preoperative PLPR (reference value 7.46), preoperative platelet count/lymphocyte count (PLR; reference value 159.56), and preoperative platelet count × NLR (SII; reference value 915.48) were related to patient prognosis (*P* < 0.05); meanwhile age, total TNM stage, preoperative PLPR (reference value 7.46), preoperative PLR (reference value 159.56), and preoperative SII (reference value 915.48) were independent hazards for prognosis (*P* < 0.05). Five independent risk factors were analyzed by nomogram model to predict the 5-year OS of patients who underwent a radical operation for carcinoma of the stomach.

**Conclusion:**

Preoperative PLPR ratio (reference value 7.46) is an independent risk factor for long-term prognosis in patients undergoing a radical operation for gastric cancer. The nomogram scoring system established by postoperative TNM staging combined with this ratio and age, PLR, and SII can better forecast the survival of patients who underwent radical operation for carcinoma of the stomach.

## 1. Introduction

The morbidity and mortality of gastric cancer locate fifth and fourth among malignant tumors, respectively [1]. Although there are many clinical treatment methods, the five-year overall survival (OS) rate of advanced gastric cancer patients undergoing radical surgery is still low. If we can find indicators to predict the long-term prognosis for clinical guidance, it will be of great significance. In 1863, the German pathologist Virchow found leukocytes in the tumor tissue and proposed that inflammation and the presence of the tumor were closely related [2]. In recent decades, tumor-related inflammation has been regarded as a key factor in cancer development, mediating tumor occurrence, proliferation, invasion, and metastasis through the release of a variety of inflammatory factors [3]. Changing neutrophil counts in the peripheral blood reflect the inflammatory state of the organism. The surveillance and clearance of tumors by the immune system mainly depend on the role of lymphocytes in the peripheral blood. If the number of lymphocytes is reduced, the immune response of tumors will be suppressed [4]. Platelets can surround tumor cells to protect them from natural killer (NK) cell killing and also can promote tumor growth, invasion, and angiogenesis [5]. Malnutrition is prevalent in cancer patients. It can destroy the body's immune system and inhibit immune function. Prealbumin can be used as one of the indicators to judge nutritional status. Currently, individual indicators, such as inflammation, immunity, coagulation, and nutrition are associated with the prognosis of gastric cancer. In some gastric cancer studies, the prognosis of groups with high neutrophil count/lymphocyte count (NLR), high platelet count/lymphocyte count (PLR), and high SII (platelet count × NLR) is poor [6–8]. Some studies have found that low serum prealbumin level is associated with poor prognosis in gastric cancer patients [9]. The relationship between preoperative platelet count/(lymphocyte count × prealbumin count) ratio (PLPR) as a combined predictor of immunity, coagulation, nutrition, and long-term survival in patients undergoing radical gastric cancer surgery has not been studied. Therefore, this study is worth exploring.

## 2. Materials and Methods

### 2.1. Retrospective Analysis

Retrospective analysis was performed on stage I–III patients who underwent radical surgery for gastric cancer in the department of gastrointestinal surgery, affiliated Cancer Hospital of Nantong University from January 2014 to June 2016. Inclusion criteria: (1) gastric cancer was confirmed by pathology; (2) patients undergoing radical gastrectomy; (3) clinical data and follow-up data were intact. Exclusion criteria: (1) patients receiving neoadjuvant chemotherapy before surgery; (2) patients with distant metastasis before surgery; (3) patients with a serious infection, blood disease, other malignant tumors, and autoimmune diseases before surgery; (4) patients with hepatitis, cirrhosis, and other serious liver diseases before operation. Finally, a total of 238 patients with gastric cancer were included.

### 2.2. Method

In this study, we performed Tumor Node Metastasis (TNM) staging in patients with gastric cancer in line with the 8th American Joint Committee on Cancer/Union International for Cancer Control. Neutrophil counts, platelet counts, lymphocyte counts, and prealbumin levels were measured within three days before surgery. Evaluation criteria include NLR, PLR, SII, and PLPR were set. According to the optimal threshold of PLPR, the distribution and correlation of various clinical indicators were analyzed, and the influence of this ratio on the 5-year OS rate was analyzed by Kaplan–Meier (K–M) curve. The Cox regression model was used to analyze the risk factors affecting the long-term survival of patients. A nomogram model was used to predict long-term survival.

#### 2.2.1. Follow-Up Methods

Enrolled patients were followed up every 3 months for the first 2 years after surgery and every 6 months thereafter until June 2021. In total, six patients were lost to follow-up. OS: the time from the date of surgery to the date of death or the date of the last follow-up.

### 2.3. Method of Statistics

SPSS 26.0 was used for statistical processing. Yoden index, Receiver operating characteristic (ROC) curve, Chi-squared test, K–M survival curve, and Cox regression model were used for the study. *P* < 0.05 was considered statistically significant.

## 3. Results

### 3.1. ROC Curve of Preoperative Peripheral Blood Parameters NLR, PLR, SII, PLPR (Associated with 5-year Overall Survival in Patients Undergoing Radical Gastric Cancer Surgery)

**Figure 1:**
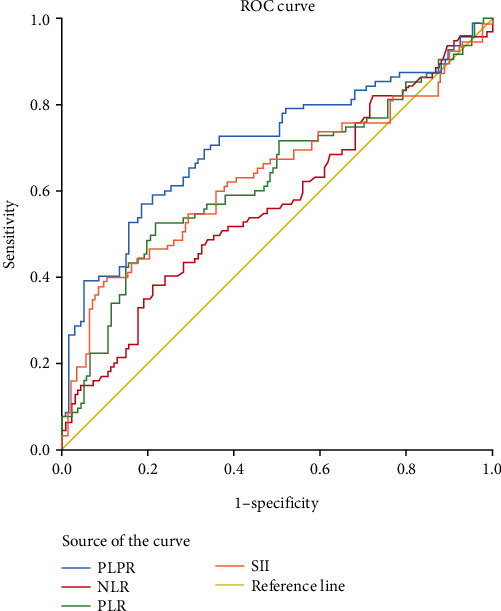
ROC curve of the preoperative NLR, PLR, SII, and PLPR (associated with 5-year OS in patients undergoing radical gastric cancer surgery).

The preoperative peripheral blood parameters NLR, PLR, SII, and PLPR were considered for the test variables, and the 5-year OS rate was considered for the status variable. The optimum cut-off point, sensitivity, and specificity were determined by the maximum Youden index. The area under the ROC curve (AUC) of PLPR was 0.711, and the optimal cut-off value corresponding to the maximum value of the Youden index was 7.46. We found that the AUC value of PLPR was significantly higher than that of the other three indexes, indicating that PLPR had better predictive efficacy than the other three indexes (see Table 1 and Figure 1 for details).

### 3.2. Clinicopathological Features of the Patients Were Included

A total of 238 patients with gastric cancer were covered in this research with an average age of 63.99 years. These included 171 males (71.85%) and 67 females (28.15%; see Table 2 for details).

### 3.3. Relationship between Preoperative PLPR and Clinicopathologic Characteristics of Patients Who Underwent Radical Operation for Carcinoma of the Stomach

The group in ratio ≥7.46 compared with the group in ratio <7.46. There was no remarkable discrepancy in gender, age, surgical method, tumor location, degree of differentiation, and pathological type (*P* > 0.05). There were prominent differences in tumor size, T stage, N stage, overall stage, vascular tumor thrombus, and nerve invasion (*P* < 0.05; see details in Table 3).

### 3.4. The Relationship between Preoperative PLPR and the Prognosis of Gastric Cancer Patients Was Evaluated by K–M Survival Curve

**Figure 2:**
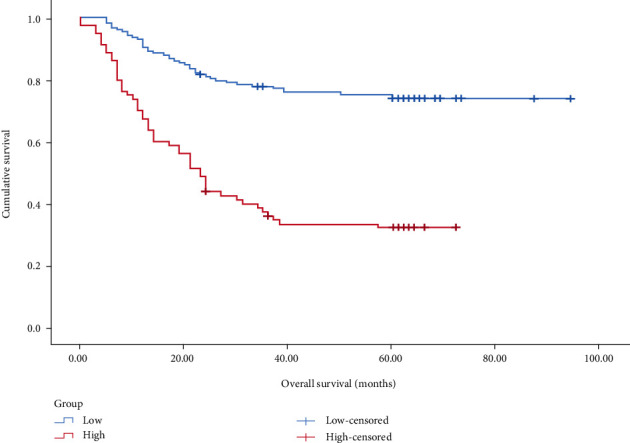
K–M curve survival analysis of 238 gastric cancer patients with high and low preoperative PLPR (*P* < 0.001). Low:preoperative PLPR **<** 7.46 and high:preoperative PLPR ≥ 7.46.

**Figure 3:**
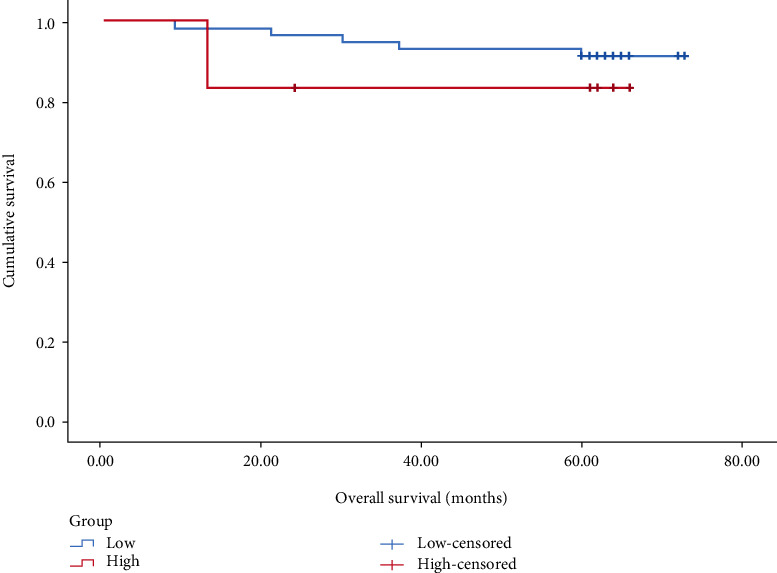
K–M survival curve analysis of stage I patients with high and low preoperative PLPR (*P* = 0.414).

**Figure 4:**
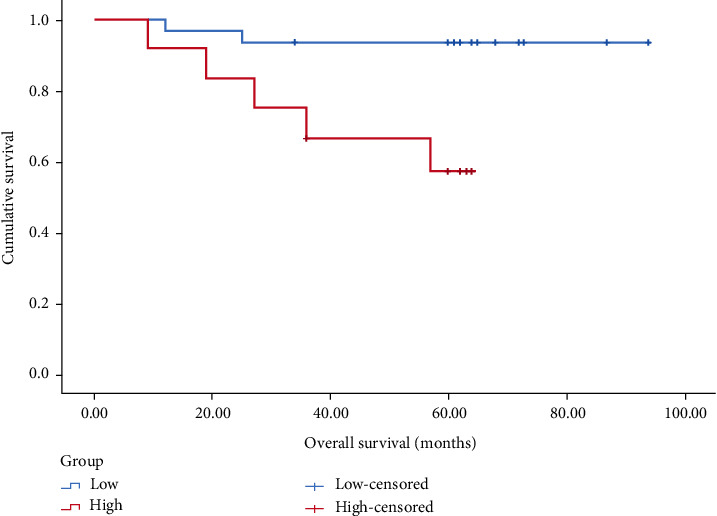
K–M survival curve analysis of stage II patients with high and low preoperative PLPR (*P* = 0.005).

**Figure 5:**
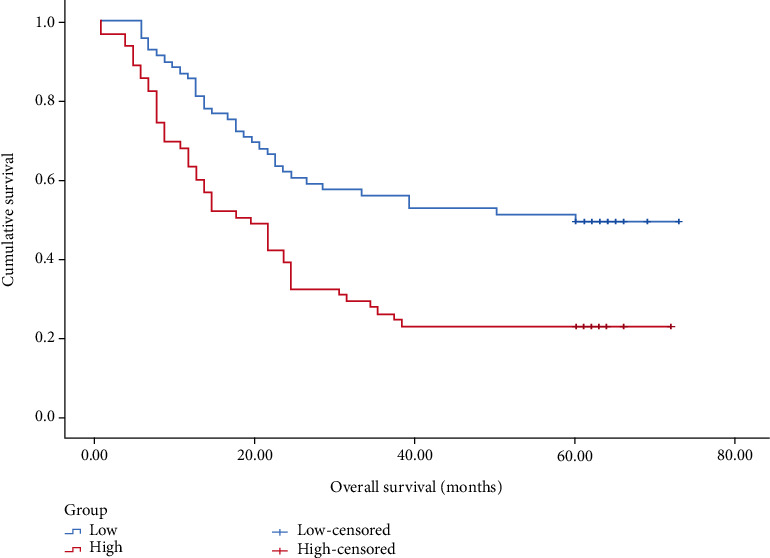
K–M survival curve analysis of stage III patients with high and low preoperative PLPR (*P* < 0.001).

In patients with stage I–III, the prognosis of the <7.46 group was significantly better than that of the prognosis of ≥7.46 group (*P* < 0.001), as shown in Figure 2. In patients with stage I, the prognosis of the <7.46 group compared with the ≥7.46 group was undifferentiated (*P* > 0.05), as shown in Figure 3. In patients with stage II, the prognosis of the <7.46 group was better than the prognosis of ≥7.46 group (*P* < 0.05), as shown in Figure 4. In patients with stage III, the prognosis of the <7.46 group was significantly better than the prognosis of ≥7.46 group (*P* < 0.001), as shown in Figure 5.

### 3.5. Cox Regression Model Was Applied to Analyze the Hazards Affecting the 5-year Overall Survival of Patients with Gastric Cancer after Radical Operation

Univariate analysis indicated that age, T stage, N stage, TNM total stage, tumor size, vascular tumor thrombus, nerve invasion, preoperative NLR (reference value: 3.68), preoperative PLPR (reference value: 7.46), preoperative PLR (reference value: 159.56), and preoperative SII (reference value: 915.48) were associated with the 5-year OS of patients (*P* < 0.05). Multivariate analysis indicated that age, TNM total stage, preoperative PLPR (reference value: 7.46), preoperative PLR (reference value: 159.56), and preoperative SII (reference value: 915.48) were independent hazards for the 5-year OS of patients (*P* < 0.05; see details in Table 4).

### 3.6. Nomogram Analysis

**Figure 6:**
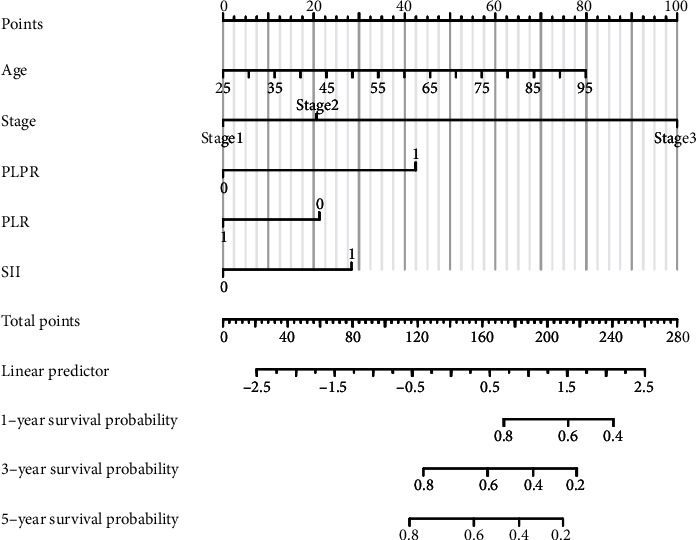
Nomogram prediction of the 5-year rate of survivors. PLPR: 0 (<7.46), 1 (≥7.46). PLR: 0 (<159.56), 1 (≥159.56), and SII: 0 (<915.48) and 1 (≥915.48).

Based on the results of multivariate Cox regression analysis, a nomogram including age, total TNM stage, preoperative PLPR (reference value: 7.46), and preoperative PLR (reference value: 159.56), and preoperative SII (reference value: 915.48) were constructed. The total score of these five independent risk factors was calculated, according to the total score, we can estimate the 5-year survival rate of patients who underwent a radical operation for carcinoma of the stomach (Figure 6).

## 4. Discussion

Gastric cancer patients are not easy to be found in the early stage, often diagnosed as locally advanced, and the long-term survival rate is low. TNM staging system, which is mainly based on postoperative pathological results, is the most commonly used indicator to forecast the prognosis of patients who had gastric carcinoma in clinical practice [10]. However, we still encounter patients whose prognosis is different from the prediction based on pathological TNM staging, emphasizing the need to combine more indicators to better predict the prognosis, which is of great significance for clinical practice to develop personalized treatment plans. There is increasing evidence that there are specific links between coagulation, immunity, nutrition, and cancer. Preoperative biomarkers in peripheral blood reflect the baseline status of patients to a certain extent and are highly accessible in clinical practice, which is taken for latent markers for forecasting prognosis [11–13]. We collected preoperative peripheral blood indexes of patients with radical gastrectomy for gastric cancer, including platelet count, lymphocyte count, and prealbumin count, and integrated these indexes to explore their relationship with the long-term prognosis of gastric cancer.

Tumor cells are removed from the primary tumor tissue and enter the bloodstream. Platelets promote the enhancement of tumor-associated coagulation, covering tumor cells by aggregating platelets and protecting them from immune attack. At the same time, platelets secrete growth factors and chemokines, inhibit the immune environment, promote tumor neovascularization, and lead to tumor proliferation and metastasis [14].

Lymphocytes are the key components of anti-tumor immunity, among which T lymphocytes are specific immune cells and play a specific anti-tumor role. B lymphocytes are effector cells of humoral immunity, which can stimulate antitumor immunity through antibody-dependent cell-mediated cytotoxicity [15, 16]. In the process of tumor development, lymphocytes in peripheral blood can migrate to the tumor microenvironment to form TILS, which plays an important role in anti-tumor immunity. Lymphopenia leads to a reduced immune response to malignant tumors and ultimately to a poorly controlled inhibitory effect on tumor proliferation [17, 18].

Inflammation promotes carcinogenesis by destroying tissues, and neutrophils play an important role in this process. Neutrophils are divided into N1 type and N2 type under the action of transforming growth factor. N1 type neutrophils increase cytotoxicity by stimulating the adaptive immune system, whereas N2 type neutrophils mainly inhibit immune responses by releasing extracellular traps, and promote tumor proliferation, metastasis, and invasion by producing cytokines and proteases [19]. In addition, a variety of enzymes and cytokines secreted by neutrophils also promote tumor development.

Malnutrition is common in patients with gastrointestinal malignant tumors, which can damage the human immune system, inhibit immune function, and lead to tumor progression. Prealbumin is synthesized by the liver and has the properties of a thymic hormone, which enhances the body's immune response by promoting the maturation of lymphocytes [20, 21]. Compared with albumin, prealbumin is more susceptible to nutritional status and its detection is more accurate. Currently, several studies have shown that decreased prealbumin is detrimental to the prognosis of patients with malignant tumors [22, 23].

In this study, we analyzed the predictive value of preoperative peripheral blood parameters NLR, PLR, SII, and PLPR for 5-year OS in patients with gastric cancer undergoing radical surgery by the area under the ROC curve, and found that the predictive value of PLPR was significantly better than the other three indicators. According to the preoperative PLPR reference value of 7.46, patients were divided into PLPR high ratio group (≥7.46) and PLPR low ratio group (<7.46). Through K–M survival curve analysis, the prognosis of patients with stage I–III gastric cancer in the low ratio group was better than that in the high ratio group. Subgroup analyses showed similar results in patients with stage II and III gastric cancer. By Cox survival analysis, we found that age, T stage, N stage, total TNM stage, tumor size, vascular tumor thrombus, nerve invasion, preoperative NLR (reference value 3.68), preoperative PLPR (reference value 7.46), preoperative PLR (reference value 159.56), and preoperative SII (reference value 915.48) were related to the 5-year OS of patients with gastric cancer after radical surgery. Age, TNM total stage, preoperative PLPR, preoperative PLR, and preoperative SII are independent risk factors for the 5-year OS of patients with gastric cancer after radical surgery. Therefore, preoperative PLPR (reference value 7.46) has good predictive power for the long-term prognosis of patients with gastric cancer after radical surgery. Compared with the patients in the low ratio group, the patients in the high ratio group had larger tumor volume, later stage, and were more likely to have vascular tumor thrombus and nerve invasion. We integrated five variables: age, total TNM stage, preoperative PLPR (reference value: 7.46), preoperative PLR (reference value: 159.56), and preoperative SII (reference value: 915.48) to construct a nomogram and establish a prognostic scoring system. Based on the TNM staging system, we further improved the prognostic scoring system, to better guide clinical practice.

This study is the first to comprehensively consider the relationship between tumor stage, pathological characteristics, coagulation, immune, nutritional status, and long-term prognosis of patients who underwent a radical operation for carcinoma of the stomach and establish a scoring system, which can guide clinical practice. However, it has certain limitations. First, it is a single-center retrospective study, the number of cases is small and there is selection bias. Second, some indicators that may be related to the research are not included, for instance, *Helicobacter pylori* and Carcinoembryonic antigen (CEA). The main reason is that these indicators are not routinely detected before surgery, resulting in the absence of some indicators. The effect of these factors needs to be further studied. Finally, we used OS as an assessment of prognosis and did not proceed with further investigation of progression-free survival(, which could also be explored in more depth.

## 5. Conclusions

The nomogram scoring system established by preoperative PLPR (reference value 7.46) combined with postoperative TNM stage, age, PLR, and SII can better forecast the prognosis of patients who underwent a radical operation for carcinoma of the stomach and guide clinical practice.

## Figures and Tables

**Table 1:** The predictive value of preoperative NLR, PLR, SII, and PLPR for the 5-year OS rate of patients undergoing radical gastric cancer surgery.

Index	AUC	*P* value	Cut-off value	Youden index	Sensitivity (%)	Specificity (%)
NLR	0.573	0.058	3.68	0.169	37.9	79.0
PLR	0.630	0.001	159.56	0.309	52.6	78.3
SII	0.634	<0.001	915.48	0.295	40.0	89.5
PLPR	0.711	<0.001	7.46	0.386	56.8	81.8

**Table 2:** Distribution of clinicopathological features.

Clinical parameters	Patients	Percentage (%)
Gender		
Female	67	28.15
Male	171	71.85
Age (years)		
<60	54	22.69
≥60	184	77.31
Surgical approach		
Open surgery	216	90.76
Laparoscopic surgery	22	9.24
Tumor location		
Cardia	34	14.29
Non-cardia	204	85.71
Differentiation		
Well/moderate	35	14.71
Poor	203	85.29
Tumor size (cm)		
<4	107	44.96
≥4	131	55.04
Pathological type		
Non-signet ring cell carcinoma	207	86.97
Signet ring cell carcinoma	31	13.03
T stage		
T1–T2	83	34.87
T3–T4	155	65.13
N stage		
N0	74	31.09
N1	37	15.55
N2	37	15.55
N3	90	37.81
TNM stage		
I–II	108	45.38
III	130	54.62
Vascular tumor thrombus		
Negative	140	58.82
Positive	98	41.18
Nerve invasion		
Negative	141	59.24
Positive	97	40.76
PLPR		
≥7.46	80	33.61
<7.46	158	66.39
NLR		
≥3.68	66	27.73
<3.68	172	72.27
PLR		
≥159.56	81	34.03
<159.56	157	65.97
SII		
≥915.48	48	20.17
<915.48	190	79.83

**Table 3:** Relationship between preoperative PLPR and clinicopathological features.

Clinicopathological parameters	PLPR		*χ* ^2^	*P*-value
	<7.46 (*n* = 158)	≥7.46 (*n* = 80)		
Gender				
Female	43	25	0.424	0.515
Male	115	55		
Age (years)				
<60	32	22	1.590	0.207
≥60	126	58		
Surgical approach				
Open surgery	143	73	0.035	0.852
Laparoscopic surgery	15	7		
Tumor location				
Cardia	26	8	1.728	0.189
Non-cardia	126	68		
Differentiation				
Well/moderate	28	7	3.408	0.065
Poor	130	73		
Tumor size (cm)				
<4	85	22	14.842	<0.001
≥4	73	58		
Pathological type				
Non-signet ring cell carcinoma	137	70	0.029	0.864
Signet ring cell carcinoma	21	10		
T stage				
T1–T2	76	7	36.210	<0.001
T3–T4	82	73		
N stage				
N0	61	13	24.763	<0.001
N1	30	7		
N2	23	14		
N3	44	46		
TNM stage				
I–II	90	18	25.447	<0.001
III	68	62		
Vascular tumor thrombus				
Negative	106	34	13.257	<0.001
Positive	52	46		
Nerve invasion				
Negative	106	35	11.981	0.001
Positive	52	45		

**Table 4:** Cox regression analysis of OS univariate and multivariate analyses.

Parameters	HR (95% CI)	*P* value	HR (95% CI)	*P* value
Sex				
Female				
Male	0.986 (0.628–1.549)	0.952		
Age (years)				
≥60				
<60	0.569 (0.328–0.988)	0.045	0.400 (0.219–0.729)	0.003
Surgical approach				
Open surgery				
Laparoscopic surgery	0.861 (0.433–1.712)	0.670		
Tumor location				
Cardia				
Non-cardia	1.227 (0.696–2.163)	0.480		
Differentiation				
Well/moderate				
Poor	0.619 (0.322–1.193)	0.152		
Tumor size (cm)				
<4				
≥4	0.262 (0.161–0.427)	<0.001	0.603 (0.351–1.034)	0.066
Pathological type				
Non-signet ring cell carcinoma				
Signet ring cell carcinoma	1.350 (0.654–2.785)	0.416		
T stage				
T1–T2				
T3–T4	0.120 (0.058–0.247)	<0.001	0.659 (0.251–1.731)	0.397
N stage				
N0–N2				
N3	0.220 (0.144–0.336)	<0.001	0.653 (0.384–1.111)	0.116
TNM stage				
I–II				
III	0.120 (0.058–0.247)	<0.001	0.414 (0.178–0.964)	0.041
Vascular tumor thrombus				
Negative				
Positive	0.288 (0.189–0.438)	<0.001	0.644 (0.390–1.063)	0.085
Nerve invasion				
Negative				
Positive	0.296 (0.194–0.450)	<0.001	0.661 (0.417–1.051)	0.080
PLPR				
<7.46				
≥7.46	0.265 (0.176–0.399)	<0.001	0.289 (0.139–0.600)	0.001
NLR				
<3.68				
≥3.68	0.542 (0.358–0.821)	0.004	1.664 (0.861–3.215)	0.130
PLR				
<159.56				
≥159.56	0.374 (0.249–0.561)	<0.001	2.180 (1.040–4.569)	0.039
SII				
<915.48				
≥915.48	0.306 (0.203–0.463)	<0.001	0.451 (0.216–0.943)	0.034

## Data Availability

Data supporting this research article are available from the corresponding author or first author upon reasonable request.
